# Efficacy and Value of Antibiotic Allergy Evaluations in a Pediatric Solid Organ Transplant Population

**DOI:** 10.1093/ofid/ofaf397

**Published:** 2025-07-01

**Authors:** Jim Liu, Timothy G Chow, Rory E Nicolaides

**Affiliations:** Department of Pediatrics, University of Texas Southwestern Medical Center, Dallas, Texas, USA; Division of Allergy and Immunology, Departments of Pediatrics and Internal Medicine, University of Texas Southwestern Medical Center, Dallas, Texas, USA; Division of Allergy and Immunology, Departments of Pediatrics and Internal Medicine, University of Texas Southwestern Medical Center, Dallas, Texas, USA

**Keywords:** allergy, antibiotic allergy delabeling, pediatric, solid organ, transplant

## Abstract

Antibiotic allergies can complicate the treatment and prophylaxis of bacterial infections in the pediatric solid organ transplant population. This single-center retrospective study revealed that antibiotic allergy evaluations and testing could safely and effectively be performed in this population group, with a positive economic value associated with sulfonamide allergy delabeling.

## Graphical Abstract

**Graphical Abstract:**
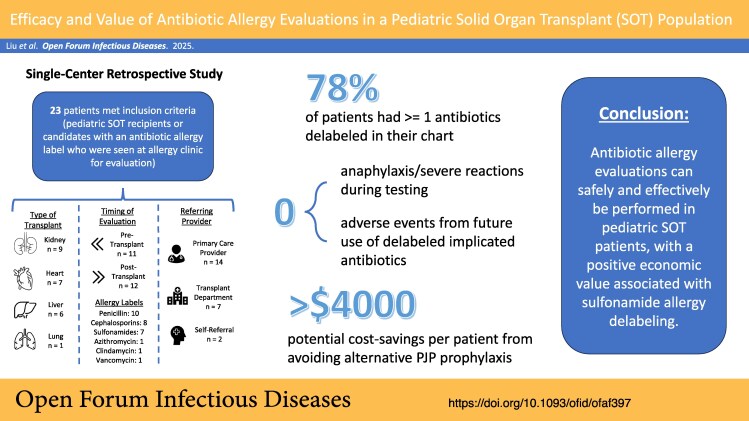

Solid organ transplants (SOTs) are standard therapy for end-stage organ failure in the pediatric population, with approximately 1800 transplants performed annually in the United States [1]. Compared to adults, pediatric transplant recipients are higher risk of developing primary infections; they may lack natural immunity if not previously exposed to certain microbes, if primary immunizations are incomplete, or if there is induced secondary immunosuppression [2]. Infections remain a major source of morbidity and mortality, and among all transplant patients, up to 63% are attributed to bacterial etiologies [3, 4]. Having a documented antibiotic allergy complicates not only the treatment of infectious complications but also limits options for prophylactic antibiotic therapy to prevent such complications in SOT patients.

Among children worldwide, up to one-third of all adverse drug reactions (ADRs) are attributed to antibiotics, the most common being penicillin [5, 6]. An allergy label refers to the addition of a medication to a patient's allergy list in the electronic medical record (EMR); the majority of labels are patient-reported and include both unpredictable immune-mediated ADRs and predictable non–immune-mediated ADRs [7]. Penicillin allergy labels (PALs) are associated with increased infection risk, length of hospital stay, and healthcare costs [6, 8–10]. Reducing erroneous PALs has been advocated from an antimicrobial stewardship perspective, with penicillin allergy testing (PAT) an effective way to delabel [11]. Despite the prevalence of PALs among children, less than 5% are confirmed to be clinically significant acute-onset immunoglobulin E–mediated allergies or delayed-onset T-cell–mediated hypersensitivities [6]. In a recent systematic review, >97% of children were delabeled following PAT, and 93% tolerated a subsequent course of β-lactam antibiotics [12]. Non–β-lactam antibiotic allergies are also prevalent, comprising 23% of reactions [13]. Sulfonamide allergies are especially disruptive to this population as they may preclude use of prophylactic regimens, like trimethoprim-sulfamethoxazole (TMP-SMX), which remains the preferred first-line agent for *Pneumocystis jirovecii* pneumonia prophylaxis at least 6–12 months after transplantation [14].

Although more than 29% of transplant patients report antibiotic allergies, few studies have examined the impact of delabeling [15]. Among adult hematopoietic stem cell transplant patients with β-lactam allergies, it was found that delabeling resulted in shorter durations of therapy and associated cost savings [16]. Another study found that 96.3% of pretransplant recipients with antibiotic allergies were safely able to receive implicated antibiotics following a delabeling intervention [17]. However, literature has been limited to adults; to our knowledge, no studies have examined antibiotic allergy evaluations within a pediatric SOT population [16, 17]. In this study, we assess the efficacy and value of antibiotic allergy evaluations within this unique population group, both before and after transplantation.

## METHODOLOGY

This study was a single-center retrospective analysis based out of Children's Medical Center in Dallas, Texas, and approved by UT Southwestern Medical Center's institutional review board (STU-2024-0299). Data were collected via chart review from Epic Systems EMR. Inclusion criteria consisted of SOT recipients or candidates with an antibiotic allergy label who were seen at Children's Medical Center Allergy Clinic for an allergy evaluation between January 2012 and October 2024. Exclusion criteria consisted of patients older than age 18 years at the time of allergy evaluation and those who had not yet completed their evaluation by the end of the study period.

Relevant patient information collected includes demographics and medical history involving implicated antibiotic(s) and adverse reaction(s) reported, time elapsed from index reaction to allergy evaluation, applicable allergy testing and/or challenge results, presence of antibiotic allergy label in chart, and use of implicated antibiotic class after evaluation. Date and type of transplant, posttransplant complications, and referring department were reviewed. For sulfonamide antibiotic delabeling, a cost analysis was conducted on transplant recipients to ascertain cost savings from avoiding alternative antimicrobial prophylaxis. Public data via www.drugs.com were referenced to determine medication costs. Duration of antimicrobial use was determined through chart review and pharmacy fills. Descriptive statistics used to describe the study population were conducted through Excel (Version 16.89.1) [Microsoft, Redmond, WA, USA].

## RESULTS

During the study, a total of 23 patients were included. Patient characteristics are described in Table 1. Mean age at evaluation was 12.1 years (standard deviation = 5.5), with 12 patients (52.2%) identifying as male. Fourteen (60.9%) antibiotic allergy evaluation referrals were via primary care provider, followed by transplant or affiliated departments and self-referrals. Hives and/or generalized rashes were the most commonly reported adverse reactions. Kidney transplants were most common, followed by heart, liver, and lung transplants. β-lactam and sulfonamide antibiotics were the most frequently reported allergy labels, with 5 patients (21.7%) having more than 1 antibiotic allergy they were evaluated for.

**Table 1.** Characteristics of Patients Who Underwent Allergy Evaluation During the Study Period

Patient Characteristics	N, %
Timing of allergy evaluation	Number of patients (N = 23)
Posttransplant	12
Pretransplant	11
Transplant type	Number of patients (N = 23)
Kidney	9
Heart	7
Liver	6
Lung	1
Referring provider specialty	Number of patients (N = 23)
PCP	14
Transplant and affiliated departments	7
Self-referral	2
Presenting clinical symptoms	Number *(*N = 23) and percentage (%) of patients^a^
Hives/generalized rash	20 (87%)
Angioedema	4 (17%)
Respiratory	3 (13%)
Not specified	1 (4%)
TEN	1 (4%)
Reported antibiotic allergy labels	Number of labels (N = 28)^b^
Penicillins	10
Cephalosporins	8
Sulfa	7
Azithromycin	1
Clindamycin	1
Vancomycin	1
Direct challenges	Number of total challenges (N = 20)
Penicillins	9
Cephalosporins	5
Sulfa	4
Azithromycin	1
Challenge outcomes	Number of negative challenges (N = 19)
Penicillins	9
Cephalosporins	4
Sulfa	4
Azithromycin	1
Delabeled antibiotics	Number of labels (N = 20)
Penicillins	10
Cephalosporins	4
Sulfa	4
Azithromycin	1
Clindamycin	1
Vancomycin	0

Abbreviations: PCP, primary care provider; TEN, toxic epidermal necrolysis.

^a^Patients could present with multiple clinical symptoms.

^b^Five patients had more than 1 antibiotic allergy that they were being evaluated for.

Five patients (21.7%) underwent penicillin skin testing, and 1 underwent delayed intradermal skin testing to cefazolin; all skin testing was negative. After evaluation, antibiotic challenge testing was recommended for 19 patients (82.6%), with 18 being successful. The 1 failed challenge was attributed to generalized pruritus after IV ceftriaxone. Eighteen patients (78.3%) had at least 1 antibiotic delabeled following evaluation, with 11 documented to have received an antibiotic of the implicated class after evaluation. No adverse events or breakthrough infections were reported in patients receiving a delabeled antibiotic. In total, 20 of 28 antibiotic labels (71.4%) were removed from patient charts. The penicillin class had the highest rate, with all 10 labels removed (100%); the cephalosporin class had the lowest, with 4 of 8 labels removed (50%).

Of 7 patients with TMP-SMX allergy labels, 3 were unable to be delabeled because of patient failure to schedule a challenge, provider failure to remove chart label, and history of toxic epidermal necrolysis. A sulfonamide delabeling cost analysis was performed for four patients who were successfully delabeled (Table 2). The calculated costs of alternative antimicrobial regimens were compared to the cost of TMP-SMX, dosed daily or 3 times per week. Alternative regimens included dapsone 100 mg daily, atovaquone 1500 mg daily, and inhaled or IV pentamidine every 28 days. Collectively, sulfonamide delabeling resulted in total cost savings between $694.20 and $16 655.24, with average savings per patient between $173.55 and $4163.81.

**Table 2.** Cost-analysis of Sulfonamide Delabeling based on Alternative PJP Prophylaxis Agents.

Prophylactic Regimen	Unit Cost	Cost for Average Duration of Use^a^	Cost Savings of TMP-SMX Daily	Cost Savings Of TMP-SMX 3x/Week
TMP-SMX 400-80 mg daily	$0.39/dose^b^	$76.05	N/A	N/A
TMP-SMX 400-80 mg 3x/week	$0.39/dose^b^	$32.59	N/A	N/A
Dapsone 100 mg daily	$1.28/dose^c^	$249.60	$173.55	$217.01
Atovaquone 1500 mg daily	$21.52/dose^d^	$4196.40	$4120.35	$4163.81
Inhaled pentamidine every 28 days	$134.03/dose^e^	$933.42	$857.37	$900.83
Intravenous pentamidine every 28 days	$125.48/dose^f^	$873.88	$797.83	$841.29

Abbreviations: PJP, *Pneumocystis jirovecii*; TMP-SMX, trimethoprim/sulfamethoxazole.

^a^195 days average duration of use after allergy evaluation, based on 4 patients who underwent successful sulfa delabeling.

^b^Unit cost based on $19.46 for fifty 400-80 mg tablets.

^c^Unit cost based on $38.52 for thirty 100 mg tablets.

^d^Unit cost based on $193.65 for 90 mL of 750 mg/5 mL oral suspension.

^e^Unit cost based on $134.03 for 1 powder for inhalation.

^f^Unit cost based on $1254.75 for 10 powders for injection.

## DISCUSSION

In this retrospective study of pediatric SOT recipients undergoing antibiotic allergy evaluations, 18 patients (78.2%) had at least 1 antibiotic delabeled in their chart following evaluation, demonstrating the efficacy of allergy evaluations within this population group. Among the 5 patients who did not have an allergy delabeled, only 1, with a history of toxic epidermal necrolysis, was due to high suspicion for a true hypersensitivity reaction. Three patients did not follow through with a challenge despite low clinical suspicion; 1 had a negative challenge, but their allergy was not removed from their chart. Thus, while our study's delabeling percentage is lower than in prior literature, modifiable factors exist to optimize delabeling rates, including proactive scheduling for challenge follow-ups and ensuring removal of allergy labels [17]. Additionally, as direct challenge testing has emerged as safe and effective in evaluating non–high-risk antibiotic allergy labels, there are increasing efforts for nonallergy specialists to perform direct challenge testing across a wide spectrum of clinical practices [18, 19]. Expanding access to direct challenge testing for low-risk antibiotic allergies in outpatient SOT clinics may reduce barriers to delabeling and maximize potential benefits; further study is needed to assess this.

Eleven of 18 patients (61.1%) with at least 1 antibiotic delabeled in their chart were documented to have received a future dose. This is partially explained by the fact that our analysis was limited to chart review within our hospital's EMR system and would not account for subsequent antibiotics given at outside institutions, especially as pediatric patients age out. All PAT was conducted in outpatient settings, and none required emergency treatment for severe allergic reactions or anaphylaxis. No adverse effects were reported for any future use of delabeled implicated antibiotics, suggesting that allergy evaluations are safe and predictive of real-world success. The low frequency of allergy evaluation referrals from transplant and affiliated departments necessitates further communication, outreach, and education to help facilitate delabeling.

Strengths of this study include patient population characteristics and inclusivity of all antibiotic allergies. The study is based at an urban specialty center, the study sample age ranges from 1 to 18 years, and 4 SOT types are represented. Compared to prior studies focusing solely on penicillin allergies, including all antibiotics allowed capture of a broad spectrum of antibiotic allergy evaluations and comparison between them. With potential cost savings found to be over $4000 per patient, sulfonamide delabeling in pediatric SOT patients also represents significant value from a healthcare expenditure perspective. Our cost analysis, albeit, is limited by a sample size of only 4 patients, in line with a general limitation of our study's smaller sample size of 23 patients. The total number of pediatric SOTs is markedly lower than adult counterparts, and as a single-center study, there exist intrinsic limitations to the number of patients. Expanding this study to a multicenter study would help increase sample size and further diversify our study population.

In conclusion, we demonstrate that antibiotic allergy evaluations can safely and effectively be performed in pediatric SOT patients, with indications of a positive economic trend associated with sulfonamide allergy delabeling. This study is novel in the population being studied and can springboard future research and interventions. Next steps include automating referral reminders for transplant patients with antibiotic allergy labels within our EMR system and establishing referral pipelines between departments.

## Notes

***Acknowledgments.*** The authors acknowledge Taylor Gheen who contributed to the development of the identification process of patients meeting inclusion criteria by providing guidance on the functionality of Epic System's SlicerDicer.

***Patient consent statement.*** Our study does not include factors necessitating patient consent.

***Financial support.*** None.
